# Temporal Patterns in Out-of-Hospital Cardiac Arrest Incidence and Outcome

**DOI:** 10.1001/jamacardio.2025.2247

**Published:** 2025-07-16

**Authors:** Owen McBride, Amy Poel, Catherine R. Counts, Megin Parayil, Camilla Osborne, Chris Drucker, Mickey Eisenberg, David Murphy, Peter Kudenchuk, Michael Sayre, Thomas Rea

**Affiliations:** 1 Department of Emergency Medicine, University of Washington, Seattle; 2 Emergency Medical Services Division, Public Health–Seattle & King County, Seattle, Washington; 3 Seattle Fire Department, Seattle, Washington; 4 Department of Medicine, Division of Cardiology, University of Washington, Seattle; 5 Department of Medicine, Division of General Medicine, University of Washington, Seattle

## Abstract

**Importance:**

Incidence and outcome of out-of-hospital cardiac arrest (OHCA) have implications for public health and community strategies to reduce risk and improve resuscitation.

**Objective:**

To examine temporal patterns in OHCA incidence and outcome.

**Design, Setting, and Participants:**

This was a retrospective cohort investigation conducted in King County, Washington, between 2001 and 2020. Adults with OHCA treated by emergency medical services (EMS) were included in the analysis. Study data were analyzed from May 2024 to April 2025.

**Exposures:**

Incidence and clinical outcome of OHCA.

**Main Outcomes and Measures:**

Annual incidence was calculated per 100 000 person-years and stratified by sex, age group (<65 years and ≥65 years), and initial rhythm (shockable, nonshockable) with change estimated as average annualized change (AAC) percentage. Resuscitation was assessed according to 5-year groups. Temporal trends were evaluated using Poisson regression for incidence and survival to hospital discharge.

**Results:**

There were 25 118 individuals (median [IQR] age, 65 [53-78] years; 15 994 male [63.7%]) with OHCA treated by EMS during 30 884 504 person-years; survival was 17.7%. Overall incidence was 81.3 per 100 000 person-years, 20.9 for shockable and 59.8 for nonshockable OHCA. There was no evidence of linear temporal change in overall incidence: 88.7 in 2001, 82.1 in 2020 (AAC, −0.5%; 95% CI, −0.9% to 0%). However, temporal patterns depended on rhythm and demographic characteristics. For example, shockable rhythm incidence declined (28.6 in 2001 and 17.9 in 2020; AAC, −2.3%; 95% CI, −2.9% to −1.5%), but change was null among nonshockable arrest (59.8 in 2001 and 63.7 in 2020; AAC, 0.3%; 95% CI, −0.1% to 0.8%). Overall survival to hospital discharge improved over time: 14.7% (859 of 5847 individuals; 2001-2005), 17.4% (1024 of 5885 individuals; 2006-2010), 19.3% (1232 of 6376 individuals; 2011-2015), and 18.9% (1322 of 7010; 2016-2020; *P* < .001 test for trend). Survival increased from 35% (591 of 1689 individuals) during the 2001 to 2005 period to 47.5% (768 of 1617 individuals) during the 2016 to 2020 period among shockable OHCA and from 6.4% (265 of 4135 individuals) during the 2001 to 2005 period to 10.1% (536 of 5323 individuals) during the 2016 to 2020 period among nonshockable OHCA (*P* < .001 tests for trend). Temporal improvement was observed in prehospital resuscitation (survival to hospital admission) and in-hospital survival (discharge among those admitted to hospital; *P* < .001 tests for trend). Outcome improvements corresponded to temporal increase in bystander cardiopulmonary resuscitation (55.5% in 2001-2005 to 73.9% in 2016-2020) and early automated external defibrillator application by non-EMS personnel (2.2% in 2001-2005 to 10.9% in 2016-2020; *P* < .001 tests for trend).

**Conclusions and Relevance:**

Results suggest that the overall OHCA incidence did not change over time, although there were differential temporal patterns among clinical subgroups. Survival improved over time overall and according to presenting rhythm, corresponding to favorable trends in community responder, prehospital, and hospital care.

## Key Points

**Question:**

Is the incidence or outcome of out-of-hospital cardiac arrest (OHCA) changing over time overall or among important demographic or clinical groups?

**Findings:**

In this cohort study including 25 118 individuals, temporal incidence patterns depended on rhythm and demographic characteristics, with some groups experiencing decrease, some increase, and others no change. In contrast, OHCA outcomes improved over time overall and among shockable and nonshockable subsets.

**Meaning:**

The results highlight the dynamic nature of OHCA incidence and resuscitation that provide a foundational context to consider strategies of OHCA prevention and treatment.

## Teaser

This cohort study investigates whether the incidence or outcome of out-of-hospital cardiac arrest (OHCA) is changing over time overall or among important demographic or clinical groups.

## Introduction

Out-of-hospital cardiac arrest (OHCA) is a significant public health challenge, claiming hundreds of thousands of lives in the US and millions of lives worldwide each year.^1,2^ Of the persons who experience OHCA, those with an initial rhythm of ventricular fibrillation (VF) have some distinct treatment strategies that specifically include early defibrillation and, in turn, are more likely to survive with favorable neurologic function than those with an initial nonshockable rhythm.^3^ Hence, the overall and rhythm-specific incidences of OHCA have implications for public health and community strategies designed to prevent OHCA and improve resuscitation.

Previously, Cobb et al^4^ reported the temporal trends in incidence of emergency medical services (EMS)–treated OHCA from 1980 to 2000 in the city of Seattle, Washington. The incidence of VF OHCA declined substantially during this time period, whereas the incidence of nonshockable OHCA remained largely unchanged, a finding replicated in other high-income regions.^5,6,7,8,9^ Overall and rhythm-specific survival was static during the 20-year time period.^4^ Investigations of OHCA incidence from more contemporary periods suggest that VF has continued to decline, although there is limited information about the historical framework, demographic-specific trends, and/or system impacts for resuscitation and clinical outcomes.^10,11,12,13,14,15,16,17^

The aim of this study was to examine temporal patterns in the incidence and outcome of EMS-treated adult OHCA in Seattle and surrounding King County, Washington, between 2001 and 2020 in order to better inform public health and EMS-based strategies for OHCA prevention and treatment.

## Methods

### Study Design, Population, and Setting

We performed a retrospective cohort investigation of all EMS-treated patients with OHCA who were 18 years and older in King County, Washington, between January 1, 2001, and December 31, 2020. Patient race and ethnicity were not systematically assessed during the study time period. The study followed the Strengthening the Reporting of Observational Studies in Epidemiology (STROBE) reporting guidelines for observational research and was approved by the University of Washington and King County Public Health Review Boards.^18^ The review boards did not require informed consent for this study because many patients die of OHCA, and the investigation was retrospective and observational. An EMS-treated patient with OHCA is defined as a person who is determined by EMS to be pulseless and receives EMS cardiopulmonary resuscitation (CPR) or a person who received a shock from an automated external defibrillator (AED) before EMS arrival. The study excluded patients 17 years or younger, those who received advanced cardiac life support (ACLS) care from an EMS agency outside King County, and those with a presumed traumatic etiology of OHCA. Seattle and King County are metropolitan regions that include urban, suburban, rural, and wilderness areas. Seattle is the most populous city, although King County has 11 other municipalities with more than 50 000 persons. The total population of King County has increased fairly steadily from approximately 1.75 million in 2001 to 2.27 million in 2020.^19^

In King County, the 2-tiered EMS system is activated by calling 9-1-1. The first tier is provided by EMT-trained firefighters who are equipped with AEDs. The second tier consists of paramedics who are activated for more serious illness including suspected OHCA and deliver ACLS including manual rhythm interpretation and defibrillation, advanced airway placement, and medication treatment via intravenous or intraosseous administration. The EMS are instructed to start resuscitation in all patients with OHCA unless there is evidence of irreversible death or the patient has a do-not-resuscitate order.^20^ The EMS system uses the American Heart Association guidelines as the foundation for resuscitation treatment approach.^21^ Patients who are resuscitated are transported to 1 of a dozen hospitals. Each hospital is equipped with 24-7 intensive care and coronary catheterization services.

### Programmatic Improvement Efforts

During the study period, there were multiple programmatic efforts aimed at improving resuscitation. With regard to bystander response, a program of telecommunicator CPR was implemented in the 1980s.^22^ A research trial of telecommunicator CPR conducted between 2007 and 2010 was associated with an increase in telecommunicator OHCA awareness, feedback, and performance, and such feedback was subsequently renewed after the trial.^23,24^ A public access AED registry was started in 1999 as a consequence of state statute supporting the bystander strategy. Subsequent investigations have observed a gradual increase in bystander AED use.^25,26^ The implementation of crowdsourcing smartphone technology to increase bystander involvement has only recently been fully implemented (after the current study period). Beginning in 2010, some law enforcement agencies were trained in CPR, equipped with AEDs, and incorporated a priority response for suspected OHCA.^27^

With regard to EMS care, EMS began to implement a high-performance CPR approach that emphasized uninterrupted CPR, expanded CPR cycles to 2 minutes, and eliminated stacked shocks beginning in 2004.^28,29^ Training and quality improvement have subsequently incorporated guideline-directed performance metrics related to compression depth, release, and rate.^21^ In 2007, hospitals provided formal plans for targeted temperature management as part of a single-site prehospital cooling trial after resuscitation.^30^ The regional EMS also participated in a single-site randomized trial of sodium nitrite.^31^ Finally, the study system participated in the Resuscitation Outcomes Consortium from 2004 to 2015. This research network required an organized infrastructure of OHCA measurement and EMS training and feedback.^32^

### Data Sources, Collection, and Definitions

The EMS system maintains a registry of each individual with OHCA treated by EMS. Information is gathered from the dispatch recording, the EMS reports, the defibrillator recording, the hospital record, and the state-issued death certificate. The information is organized according to the Utstein data definitions.^33^ The presenting OHCA rhythm is determined through review of the defibrillator ECG recording, a methodology consistent over the study period. The registry achieves 99.8% completion of outcome status and is the basis for prospective research.^32^

### Outcomes

We determined the incidence of EMS-treated OHCA as the count of events per 100 000 population per year. The person-years value was based on the average population for each study year. Resuscitation outcomes included admission to the hospital, survival to hospital discharge, and favorable neurological survival defined as Cerebral Performance Category (CPC) 1 or 2 at hospital discharge.^33^ Briefly, CPC of 1 is defined as normal, 2 as disability but independent, 3 as disability and dependent, and 4 as unconscious.^34^ As a secondary outcome that combines incidence and outcome, we also assessed *survivor incidence*, defined as the number of survivors per 100 000 persons population per year.

### Statistical Analysis

To estimate annual OHCA incidence, we used publicly available information published annually by the Washington State Office of Financial Management to determine population size and demographic characteristics of the King County population. Because age and sex are strong risk factors for OHCA, incidence was calculated overall and for presenting OHCA rhythm (shockable vs nonshockable) according to sex and age groups: 18 to 64 years and 65 years and older. Within each category, change in incidence over time was analyzed with year modeled continuously using joinpoint trend analysis software and reported as the average annualized change (AAC) percentage over the 20-year period. The significance of a linear trend in incidence over time was determined using the Mantel-Haenszel test for linear association modeling year as a continuous variable (2001 through 2020). A secondary analysis determined if the addition of an inflection point (change in slope at a given calendar year) improved data fit for the 20-year study period.

To evaluate temporal trends in resuscitation, we determined the circumstances, presentation, care, and outcome of patients with EMS-treated OHCA according to 5-year time periods: 2001 to 2005, 2006 to 2010, 2011 to 2015, and 2016 to 2020 and tested for trend over time using the 2001 to 2005 group as the referent. We used Poisson regression to evaluate the association between time period and outcome after adjustment for age and sex and adjustment for age, sex, witness status, OHCA location, and initial OHCA rhythm. Resuscitation outcome analyses were also stratified by initial OHCA rhythm: shockable vs nonshockable. We also report characteristics and outcomes according to location (residential, public) and age group (<65 years, ≥65 years). Finally, we plotted survival and CPC 1-2 survival according to 2-year time periods alongside corresponding programmatic and research initiatives. Statistical significance was defined as 2-sided *P* < .05 for all analyses. Data were analyzed from May 2024 to April 2025 using R software, version 4.4 (R Project for Statistical Computing).

## Results

### Population

**Figure 1.**
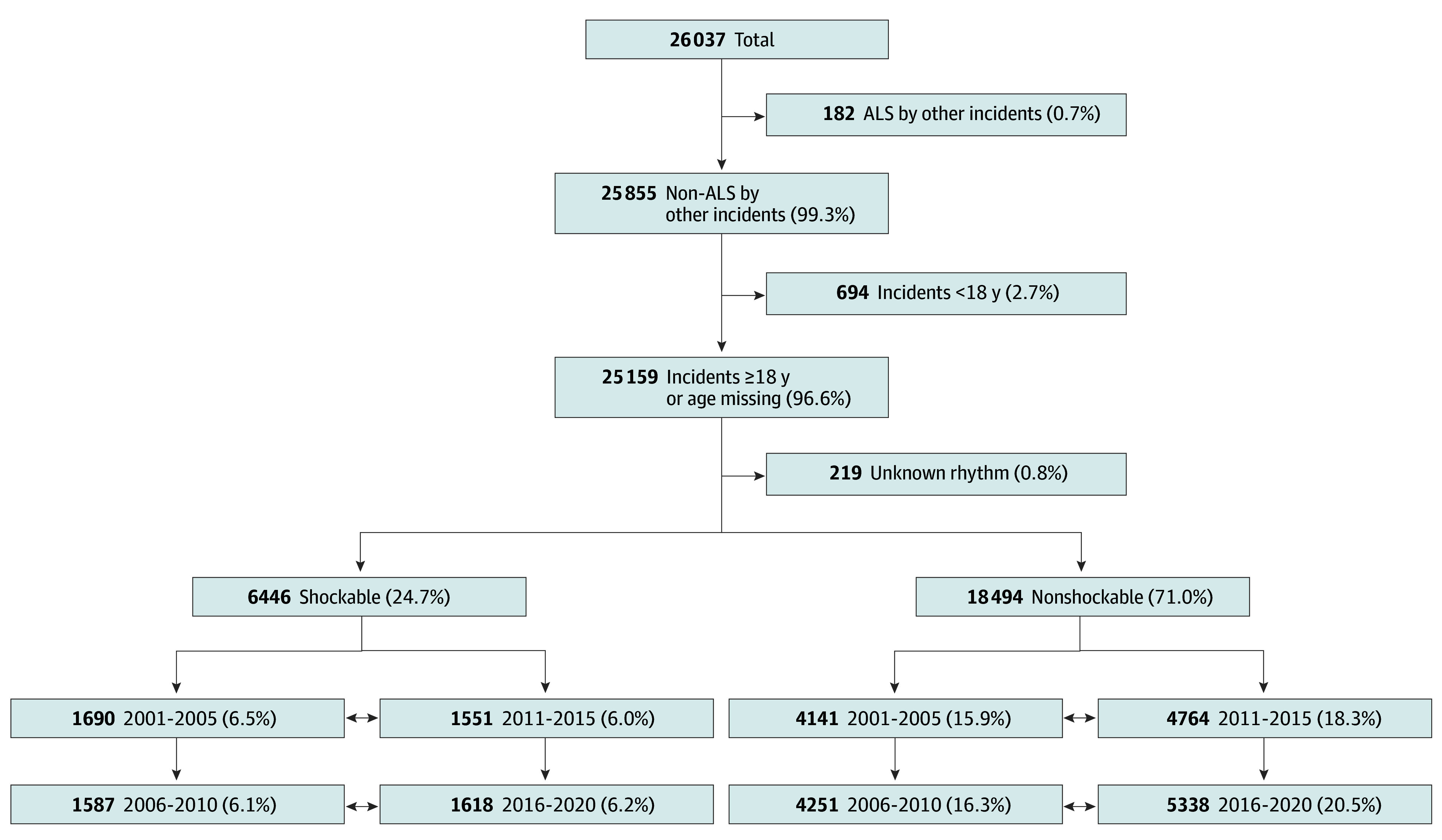
Flow Diagram According to Initial Rhythm and Calendar Year Group Study population flow diagram according to initial out-of-hospital cardiac arrest (OHCA) rhythm and calendar year group. Percentages are out of the total. ALS indicates advanced life support.

The total number of individuals with EMS-treated OHCA during the 20-year study period was 26 037. Of these, 694 were a priori excluded because of age 17 years or younger, and 182 were excluded because they were primarily treated by EMS agencies outside of King County (Figure 1). Of the eligible 25 118 individuals (median [IQR] age, 65 [53-78] years; 9124 female [36.3%]; 15 994 male [63.7%]) with OHCA, 6442 presented with shockable rhythm,18 464 presented with nonshockable rhythm, and 212 had an undocumented presenting rhythm. Overall survival was 17.7%.

### Incidence

During the 20-year period, there were 25 118 OHCAs during 30 884 504 person-years, producing an overall incidence of EMS-treated OHCA of 81.3 per 100 000 person-years, 20.9 for shockable OHCA and 59.8 for nonshockable OHCA. Risk was greater among males than females: 104.5 per 100 000 person-years for males and 58.6 per 100 000 person-years for females and was greater among the older age group: 294.4 per 100 000 person-years for those 65 years and older and 45.5 per 100 000 person-years for those aged 18 to 64 years.

### Temporal Patterns of Incidence Overall and According to Rhythm, Age, and Sex

**Figure 2.**
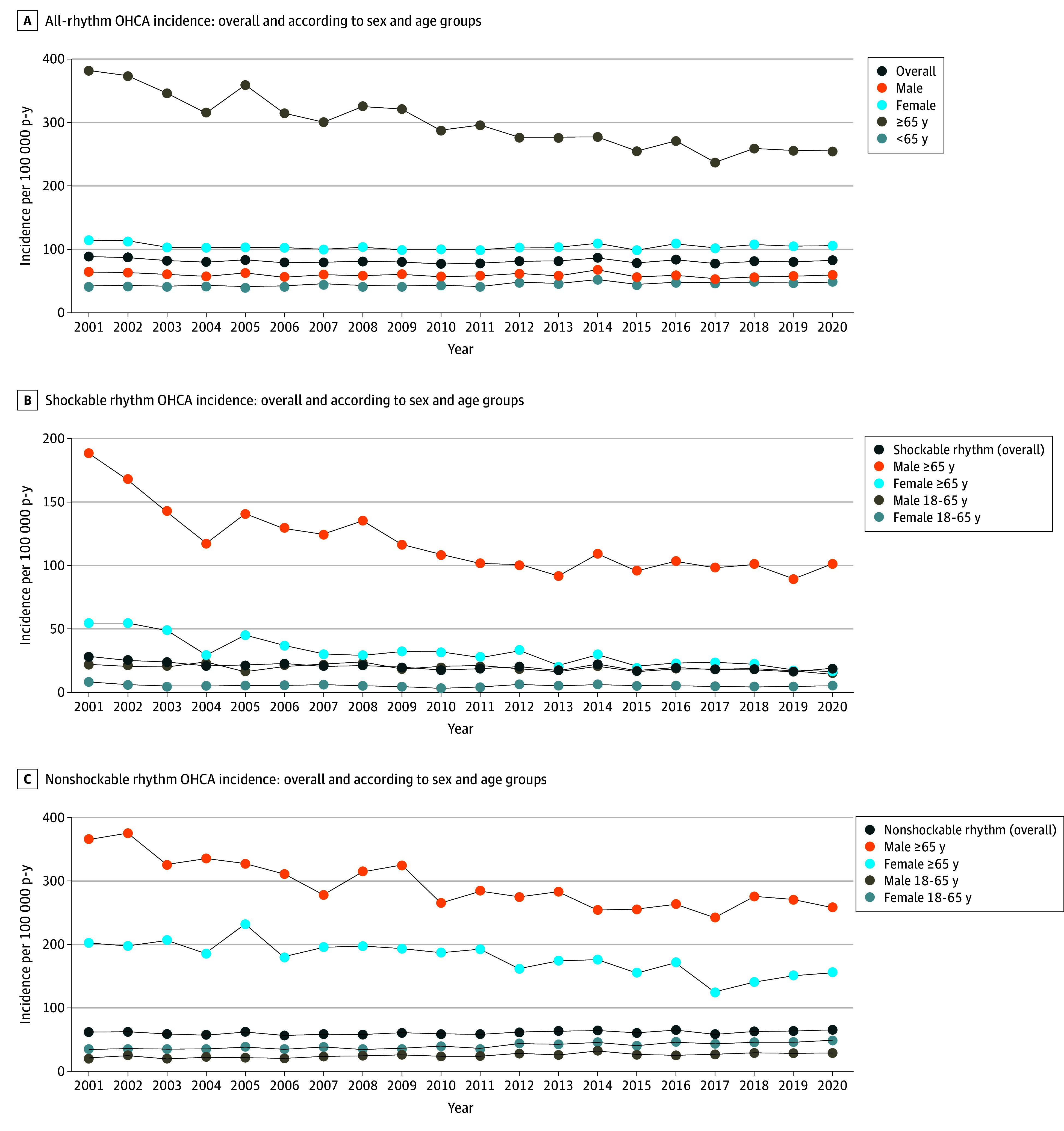
Incidence of Out-of-Hospital Cardiac Arrest (OHCA) Over Time A, All-rhythm OHCA incidence: overall and according to sex and age groups. B, Shockable rhythm OHCA incidence: overall and according to sex and age groups. C, Nonshockable rhythm OHCA incidence: overall and according to sex and age groups.

The counts of OHCA according to calendar year, presenting rhythm, age group, and sex are provided in eTable 1 in Supplement 1. There was no evidence of linear temporal change in overall incidence. Overall incidence of OHCA was 88.7 in 2001 and 82.1 in 2020 per 100 000 person-years (AAC, −0.5%; 95% CI, −0.9% to 0%) (Figure 2). The temporal trend in incidence depended on demographic characteristics and OHCA rhythm. Incidence over time did not change among males (114.8 in 2001 to 106.7 in 2020; AAC, −0.5%; 95% CI, −0.8% to 0.1%) or females (63.3 in 2001 to 57.6 in 2020; AAC, −0.4%; 95% CI, −0.9% to 0.1%). Incidence declined among older adults 65 years and older (382.9 in 2001 to 257.4 in 2020; AAC, −2.2%; 95% CI, −2.7% to −1.6%) but increased among those aged 18 to 65 years (43.2 in 2001 to 49.4 in 2020; AAC, 0.9%; 95% CI, 0.5%-1.4%) (Figure 2A). Incidence of shockable rhythm declined over time (28.6 in 2001 to 17.9 in 2020; AAC, −2.3%; 95% CI, −2.9% to −1.5%), but the change over time was null among nonshockable arrest (59.8 in 2001 to 63.7 in 2020; AAC, 0.3%; 95% CI, −0.1% to 0.8%) (Figure 2B and C).

Models used joinpoint methods to evaluate whether the association between incidence and calendar year was improved by incorporating a change in slope over the 20-year period. Such models observed that the fit of incidence vs calendar year was improved with the addition of a second slope among all arrests, among shockable arrest, and among male individuals. Specifically, models demonstrated an initial marked decline in incidence of VF and OHCA in males during years 2001 to 2004, but then a null slope between 2005 to 2020 (eTable 2 in Supplement 1). In contrast, the model of incidence vs calendar year did not improve with the addition of a change in slope among female individuals, among nonshockable arrest, or among younger or older age groups.

### Temporal Patterns of Rhythm-Specific Incidence Stratified by Age and Sex

Among those presenting with shockable OHCA, incidence declined over time among older males (189.8 in 2001 to 100.1 in 2020 per 100 000 person-years; AAC, −3.8%; 95% CI, −4.4% to −2.8%), older females (56.2 to 17.1; AAC, −5.4%; 95% CI, −6.8% to −4.0%), younger males (22.8 to 16.2; AAC, −1.3%; 95% CI, −2.0% to −0.7%), and among younger females (8.6 to 5.9; AAC, −2.1%; 95% CI, −3.7% to −0.5%) (Figure 2B). Among nonshockable OHCA, incidence declined over time among male and female individuals 65 years and older: male (366.3 in 2001 to 258.9 in 2020; AAC, −1.6%; 95% CI, −2.3% to −1.0%) and female (201.5 in 2001 to 155.4 in 2020; AAC, −2.0%; 95% CI, −2.8% to −1.2%) but increased among male and female individuals younger than 65 years: male (35.5 in 2001 to 48.8 in 2020; AAC, 1.8%; 95% CI, 1.2%-2.4%) and female (18.9 in 2001 to 27.1 in 2020; AAC, 1.7%; 95% CI, 1.0%-2.5%) (Figure 2C).

### Resuscitation Outcomes

Over the 20-year study period, 17.7% survived to hospital discharge, and 15.3% survived with favorable neurological status (Table 1). Overall survivor incidence was 14.4 per 100 000 population person-years. When stratified by initial rhythm, those with a shockable rhythm compared with nonshockable rhythm were on average more likely to be younger, male, have a cardiac etiology, have a witnessed event, receive bystander CPR, have an AED applied before EMS, and receive hospital-based targeted temperature management and coronary angiography (eTable 3 in Supplement 1). Survival to hospital discharge was 42.6% among those presenting with a shockable rhythm and 8.8% among those presenting with a nonshockable rhythm.

**Table 1.** Cardiac Arrest Resuscitation Characteristics: Overall and According to Time Period (N = 25 118)^a^

Characteristics	Overall				
	2001-2020 (n = 25 118)	2001-2005 (n = 5847)	2006-2010 (n = 5885)	2011-2015 (n = 6376)	2016-2020 (n = 7010)
Age, median (IQR), y	65 (53-78)	68 (54-80)	66 (53-80)	65 (53-78)	64 (52-75)
Sex, No. (%)					
Female	9124 (36.3)	2148 (36.7)	2164 (36.8)	2377 (37.3)	2435 (34.7)
Male	15 994 (63.7)	3699 (63.3)	3721 (63.2)	3999 (62.7)	4575 (65.3)
Cardiac etiology, No. (%)	16 860 (67.1)	4248 (72.7)	4061 (69.0)	4297 (67.4)	4254 (60.7)
Location of arrest					
Home, No. (%)	15 846 (63.1)	3668 (62.7)	3629 (61.7)	4021 (63.1)	4528 (64.6)
Public indoor/outdoor, No. (%)	4495 (17.9)	943 (16.1)	1025 (17.4)	1210 (19.0)	1317 (18.8)
Health care facility, No. (%)	429 (1.7)	88 (1.5)	94 (1.6)	87 (1.4)	160 (2.3)
Other, No. (%)	4348 (17.3)	1148 (19.6)	1137 (19.3)	1058 (16.6)	1005 (14.3)
Shockable initial rhythm, No. (%)	6442 (25.6)	1689 (28.9)	1586 (26.9)	1550 (24.3)	1617 (23.1)
Witnessed					
Bystander witnessed, No. (%)	10 167 (40.5)	2307 (39.5)	2311 (39.3)	2643 (41.5)	2906 (41.5)
EMS witnessed, No. (%)	2901 (11.5)	634 (10.8)	687 (11.7)	711 (11.2)	869 (12.4)
Bystander CPR, No. (%)^b^	14 479 (65.2)	2891 (55.5)	3178 (61.1)	3870 (68.3)	4540 (73.9)
Non-EMS AED application^b^					
AED application, No. (%)	1375 (6.2)	113 (2.2)	199 (3.4)	391 (6.9)	672 (10.9)
AED applied by law enforcement, No. (%)^c^	570 (2.6)	NA	1 (0)	160 (2.8)	409 (6.7)
AED applied by non–law enforcement, No. (%)^c^	467 (2.1)	NA	42 (0.8)	165 (2.9)	260 (4.2)
Arrest occurred before EMS arrival, No. (%)	22 198 (88.4)	5210 (89.1)	5195 (88.3)	5656 (88.7)	6137 (87.5)
BLS response time, median (IQR)^d^	5.0 (4.0-6.5)	5.0 (4.0-6.1)	5.0 (4.0-6.0)	5.0 (4.0-6.2)	5.5 (4.4-6.9)
ALS response time, median (IQR)^d^	9.0 (7.0-12.1)	9.0 (7.2-12.0)	9.0 (6.9-12.0)	8.9 (6.7-11.8)	9.7 (7.3-12.9)
Prehospital interventions					
Advanced airway, No. (%)	21 375 (85.1)	4888 (83.6)	5045 (85.7)	5395 (84.6)	6047 (86.3)
Epinephrine use, No. (%)	18 788 (74.8)	3834 (65.6)	4552 (77.3)	4893 (76.7)	5509 (78.6)
Prehospital outcomes					
Hospital admission with spontaneous pulse, No. (%)	10 124 (40.3)	2100 (35.9)	2375 (40.4)	2686 (42.1)	2963 (42.3)
Hospital interventions^e^					
Targeted temperature management, No. (%)	4009 (39.6)	216 (10.3)	1171 (49.3)	960 (35.7)	1662 (56.1)
Angiography, No. (%)	2809 (27.7)	458 (21.8)	734 (30.9)	588 (21.9)	1029 (34.7)
PCI, No. (%)	1397 (13.8)	263 (12.5)	342 (14.4)	280 (10.4)	512 (17.3)
Overall outcomes					
Survival to hospital discharge, No. (%)	4437 (17.7)	859 (14.7)	1024 (17.4)	1232 (19.3)	1322 (18.9)
Survival with CPC ≤2, No. (%)	3836 (15.3)	700 (12.0)	863 (14.7)	1086 (17.0)	1187 (16.9)
Survival with CPC ≤2 among survivors, No. (%)	3836 (86.5)	700 (81.8)	863 (84.4)	1086 (88.6)	1187 (90.1)
Survivor incidence (per 100 000 person-years)	14.4	12.3	13.8	15.7	15.2
Survivor incidence with CPC ≤2 (per 100 000 person-years)	12.4	10.1	11.6	13.9	13.7

Abbreviations: AED, automated external defibrillator; ALS, advanced life support; BLS, basic life support; CPC, Cerebral Performance Category; CPR, cardiopulmonary resuscitation; EMS, emergency medical services; NA, not available; PCI, percutaneous coronary intervention.

^a^ Each characteristic and outcome demonstrated a significant test for trend with *P* < .05.

^b^ Only patients that arrested before EMS arrival were eligible for bystander CPR and non-EMS AED application.

^c^ AED application stratified by law enforcement vs public access information available as of 2007.

^d^ Response time is from 911 call to BLS/ALS arrival at scene.

^e^ Denominator is cases with hospital admission.

### Temporal Patterns of Resuscitation Care and Outcome

Although there were absolute differences in the Utstein characteristics between shockable and nonshockable rhythms, there were common temporal patterns across the 2 rhythm groups with regard to circumstances, care, and outcome (Table 1). Average age became younger over time (68 years during 2001-2005 and 64 years during 2016-2020; *P* < .001). We observed a temporal increase in the proportion who had a noncardiac etiology, witnessed arrest, received bystander CPR, had AED application before EMS, presented with nonshockable rhythm, and received targeted temperature management, coronary angiography, and percutaneous coronary intervention (*P* < .001 for test of trend for each characteristic). EMS response intervals increased modestly but significantly over the 20-year period (5.0 minutes during 2001-2005 and 5.5 minutes during 2016-2020; *P* < .001).

**Figure 3.**
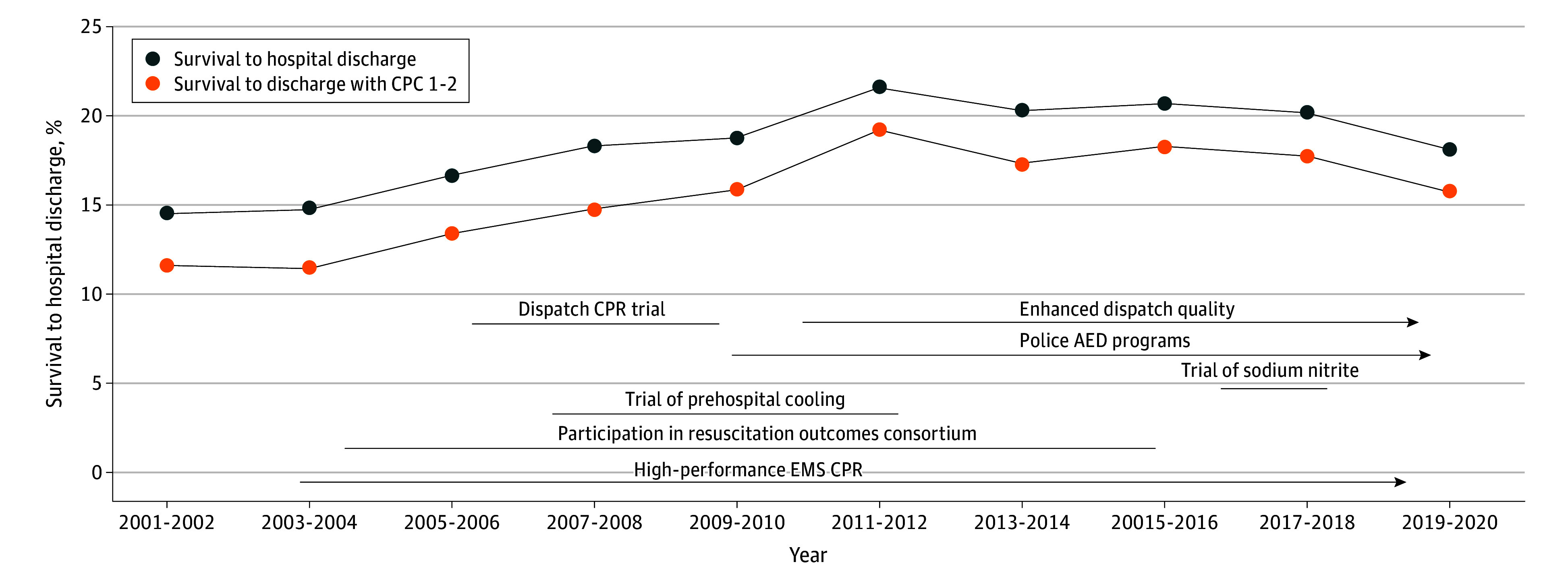
Survival Over Time (Seattle and King County) AED indicates automated external defibrillator; CPC, Cerebral Performance Category; CPR, cardiopulmonary resuscitation; EMS, emergency medical services.

With regard to outcomes, we observed a temporal improvement in survival to hospital admission, hospital discharge, and survival with favorable functional status overall and among presenting rhythm subgroups. All-rhythm survival was 14.7% during 2001 to 2005, 17.4% during 2006 to 2010, 19.3% during 2011 to 2015, and 18.9% during 2016 to 2020 (*P* < .001 test for trend across the 5-year groups), although there was no evidence of outcome improvement between the final 2 time periods. We, likewise, observed a temporal increase in survivor incidence, increasing from 12.3 survivors per 100 000 person-years in 2001 to 2005 to 15.2 survivors per 100 000 person-years in 2016 to 2020 (Table 1). The temporal improvement in overall outcomes persisted after adjustment for demographic and circumstances characteristics and was evident for prehospital and in-hospital outcomes (Table 2 and Figure 3), although again, there was no difference when comparing the final 2 time periods. Among shockable OHCA, survival increased from 35% during the 2001 to 2005 period to 47.5% during the 2016 to 2020 period (*P* < .001 test for trend) (eTables 3 and 4 in Supplement 1). Among nonshockable OHCA, survival increased from 6.4% during the 2001 to 2005 period to 10.1% during the 2016 to 2020 period (*P* < .001 test for trend) (eTables 3 and 5 in Supplement 1). Temporal patterns were generally similar according to location and age group (eTables 6-9 in Supplement 1).

**Table 2.** Relative Risk (RR) of Out-of-Hospital Cardiac Arrest (OHCA) Outcome According to Time Period

Time period	Crude %	Model 1 (crude)		Model 2^a^			Model 3^b^		
		RR (95% CI)	*P* value for trend	Adjusted, %	RR (95% CI)	*P* value for trend	Adjusted, %	RR (95% CI)	*P* value for trend
**Overall, survival to hospital discharge**									
2001-2005	14.7	1 [Reference]	<.001	14.7	1 [Reference]	<.001	14.7	1 [Reference]	<.001
2006-2010	17.4	1.18 (1.09-1.29)		17.2	1.17 (1.08-1.27)		17.5	1.19 (1.10-1.28)	
2011-2015	19.3	1.32 (1.21-1.42)		18.8	1.28 (1.19-1.39)		19.6	1.33 (1.24-1.43)	
2016-2020	18.9	1.28 (1.19-1.39)		17.9	1.22 (1.13-1.32)		19.1	1.30 (1.22-1.40)	
**Overall, survival to discharge with CPC 1 or 2**									
2001-2005	12.0	1 [Reference]	<.001	12.0	1 [Reference]	<.001	12.0	1 [Reference]	<.001
2006-2010	14.7	1.22 (1.12-1.34)		14.5	1.21 (1.10-1.33)		14.8	1.23 (1.13-1.33)	
2011-2015	17.0	1.42 (1.30-1.55)		16.7	1.39 (1.27-1.51)		17.3	1.44 (1.33-1.56)	
2016-2020	16.9	1.41 (1.30-1.54)		16.0	1.33 (1.22-1.46)		17.3	1.44 (1.33-1.55)	
**Prehospital outcome, survival to hospital admission**									
2001-2005	35.9	1 [Reference]	<.001	35.9	1 [Reference]	<.001	35.9	1 [Reference]	<.001
2006-2010	40.4	1.12 (1.07-1.18)		40.2	1.12 (1.10-1.17)		40.6	1.13 (1.08-1.18)	
2011-2015	42.1	1.17 (1.12-1.23)		41.6	1.16 (1.11-1.22)		42.0	1.17 (1.12-1.22)	
2016-2020	42.3	1.18 (1.13-1.23)		41.6	1.16 (1.11-1.21)		42.0	1.17 (1.12-1.22)	
**In-hospital outcome, survival with CPC 1 or 2 among those admitted to hospital**									
2001-2005	33.3	1 [Reference]	<.001	33.3	1 [Reference]	<.001	33.3	1 [Reference]	<.001
2006-2010	36.3	1.07 (0.98-1.16)		35.0	1.05 (0.97-1.14)		37.0	1.11 (1.03-1.20)	
2011-2015	40.4	1.20 (1.11-1.29)		38.6	1.16 (1.07-1.25)		41.6	1.25 (1.16-1.33)	
2016-2020	40.1	1.18 (1.09-1.28)		37.3	1.12 (1.04-1.21)		40.6	1.22 (1.14-1.31)	

Abbreviation: CPC, Cerebral Performance Category.

^a^ Model 2 adjusted for age and sex.

^b^ Model 3 adjusted for age, sex, initial rhythm, witnessed status, and location of arrest.

## Discussion

In this population-based cohort investigation of OHCA, we evaluated temporal patterns in incidence and resuscitation outcomes. There was no temporal change in incidence, although this lack of change in overall incidence belies differential trends according to arrest rhythm and age group. OHCA incidence declined over time among shockable rhythms and older adults (≥65 years), did not change among nonshockable rhythms, and actually increased among younger adults (18-65 years). Resuscitation outcomes improved over time, a temporal trend that was evident overall and when stratified by presenting arrest rhythm. The outcome improvements corresponded to improvements in health services such as increase in bystander CPR, AED application before EMS among patients with shockable rhythm, and hospital-based care with targeted temperature management and coronary intervention. The results demonstrate the dynamic nature of OHCA incidence and resuscitation care and outcome that collectively help provide a foundational context to consider strategies of prevention and treatment.

### Overall Incidence

The lack of temporal decline in overall OHCA incidence highlights the challenges of cardiac arrest prevention, appreciating that most OHCA occurs among individuals who are not identified as highest risk.^35^ Given that OHCA is often a consequence of complications of acute and/or chronic cardiac disease, improvements in lifestyle, medication, or interventional advances that prevent or treat heart disease can contribute to the downward trends in OHCA incidence. During this time period, temporal patterns in risk factors likely had differential incidence effects. Beneficial developments in tobacco use, lipid lowering, coronary revascularization, and implanted cardioverter-defibrillator placement were countered to some extent by increasing prevalence of obesity, hypertension, and diabetes.^1^

### Temporal Patterns in Incidence Among Demographic and Clinical Subgroups

Indeed, the null overall temporal change in incidence comprised distinct temporal trends across demographic characteristics, presenting arrest rhythm, or potentially time periods. The incidence of OHCA decreased among older adults, which is a group who are more likely to have clinically recognized cardiovascular disease and, hence, receive preventive treatments.^36^ In contrast, younger adults have a lower absolute OHCA risk but experienced a temporal increase in risk, perhaps because they have lower rates of preventive treatments and/or are disproportionately affected by noncardiac risks such a substance use disorder, which can culminate in OHCA.^37,38^

We also observed differential temporal patterns when comparing incidence according to presenting OHCA rhythm. On average, the incidence of VF declined 2.3% per year, a relative decline that is comparable with that observed during the time period of 1980 to 2000 originally reported by Cobb et al.^4^ The downward trend was observed among younger and older adults and among male and female individuals. However, the temporal decline in shockable incidence appeared to be driven by a marked decrease early on (2001-2005) with no additional decrease over subsequent years. In contrast, the incidence of nonshockable rhythms did not change over time. Nonshockable OHCA has a more heterogeneous collection of etiologies (ie, overdose, respiratory, cardiovascular, sepsis) that have not necessarily experienced the same temporal improvements in prevention and/or treatment.^37,38,39,40^

### Outcomes

The proportion of arrests presenting with a shockable rhythm declined from 25% to 20% during the 20-year study period. Despite the increasing predominance of nonshockable OHCA, the majority of survivors originated from the shockable subset even in the most recent 5-year time period, where 60% of survivors presented with VF and 40% presented with nonshockable rhythm.^41^ These results highlight the need for efforts to consider resuscitation strategies that incorporate shockable and nonshockable rhythms to address the public health challenge of OHCA resuscitation.

### Temporal Patterns of Survival

Over the 20-year period, nearly 4500 persons survived OHCA, most with good functional status. These 4500 persons translate to a survivor incidence of approximately 14 survivors to hospital discharge and 12 survivors to discharge with good neurological function per 100 000 person-years of population, highlighting the public health relevance of a strong resuscitation system of care. Importantly, functional survival improved over time overall and among rhythm-specific OHCA, suggesting that both rhythm groups are amenable to outcome improvements. This improvement persisted after accounting for temporal changes in demographic and circumstance characteristics. Moreover, temporal improvement was observed in both prehospital resuscitation (survival to hospital admission) and in-hospital survival (discharge among those admitted to hospital). The trend was evident despite the challenge of the COVID-19 pandemic that saw survival decline globally and locally during the final year of the study period.^42,43^ Indeed, the temporal trend in outcome improvement was largely driven by stepwise improvement over the first three 5-year time periods as there was no outcome difference between the final 2 time periods.

Changes in evidence-based clinical care—supported by programmatic implementation—corresponded to the observed temporal survival improvement.^44^ The study system achieved increases in bystander CPR (due in part to telecommunicator CPR), early AED application (due to police and public AED programs), and hospital care involving targeted temperature management and coronary care. Other temporal changes that reflect the quality of EMS (CPR metrics) and hospital care (withdrawal of care) may have also contributed to temporal improvement, although systematic measurement of these metrics throughout the course of the study is incomplete. For example, prior investigation from the study system suggests temporal improvement in the quality of EMS CPR.^28,29,45^ Importantly, the temporal survival increase occurred in a system with good baseline care and outcomes, providing support that resuscitation quality improvement efforts can continue to increase beneficial public health impacts even in higher-performing systems.

Efforts aimed at improvement require consistent measurement to objectively assess progress. Beyond this foundational tenet, there are a host of resuscitation improvement initiatives that translate science into community action and clinical practice. In the current study, there were positive changes in evidence-based care involving bystanders, law enforcement, EMS, and hospitals, highlighting the range of opportunity and real-world process by which these programs interface to improve survival. Importantly, time is a powerful outcome predictor such that efforts designed to reduce time to care especially for the early links that involve active resuscitation are well positioned to have public health impact. To this point, there was a marked increase in bystander CPR and early defibrillation. Initiatives such as the Global Resuscitation Alliance emphasize real-world implementation and improvement for a wide range of systems, understanding that resources, operational infrastructure, clinical expertise, and interorganizational collaboration differ substantially across systems.^46,47^ Other initiatives such as school training and AED placement can be influenced by legislative requirements.^48,49^

### Limitations

This study has limitations. Although derived from a relatively large, well-characterized population, the investigation is a singular regional experience that could affect the generalizability as OHCA incidence and outcome can vary based on geography.^50^ Moreover, some persons who experience OHCA have an emergency response but do not receive a resuscitation attempt because the person has signs of irreversible death or a do-not-resuscitate order. There are also some OHCA events that do not have a 9-1-1 medical response at all.^20^ Importantly, the approach to ascertain OHCAs, determine the stratification variables (age, sex, initial rhythm), and measure prehospital and hospital resuscitation care was consistent over time, although important characteristics related to quality (ie, CPR performance metrics) and timing (ie, withdrawal of care) were not systematically available for the 20-year study period. The research did not assess the profile of OHCA risk factors in the community and instead relied on other investigations to help inform temporal developments in OHCA risk factors. Finally, the observed temporal associations of resuscitation treatments do not establish causality with regard to survival improvements. Hence, the observed trends do not establish causality. Nonetheless, the rigorous methods provide useful insight about overall and group-specific incidence and underscore health services elements that correspond to temporal outcome improvement in this large metropolitan system.

## Conclusions

In this population-based cohort investigation of OHCA, results suggest that there was no temporal trend in overall OHCA incidence, although there were differential temporal patterns among subgroups defined by age and initial rhythm with incidence declining for shockable rhythm and older adults, not changing among nonshockable rhythms, and increasing among younger adults. Survival improved over time overall and according to presenting rhythm, corresponding to favorable trends in community responder, prehospital, and hospital health services. Collectively, the findings underscore the ongoing public health challenge of OHCA and support the need to advance prevention and resuscitation strategies through rigorous science and evidence-based implementation.

## Notes

Supplement 1. **eTable 1.** OHCA Counts and Population Denominators According to Demographics and Initial Rhythm (King County, WA 2001-2020) **eTable 2.** Joinpoint Where Second Slope Term Improves Incidence vs Calendar Year Fit **eTable 3.** OHCA Resuscitation Characteristics According to Initial Rhythm and Time Period **eTable 4.** Relative Risk of Outcomes According to Time Period for Shockable Rhythm OHCA **eTable 5.** Relative Risk of Outcomes According to Time Period for Nonshockable Rhythms OHCA **eTable 6.** OHCA Resuscitation Characteristics According to Time Period for Home Location **eTable 7.** OHCA Resuscitation Characteristics According to Time Period for Public Location **eTable 8.** OHCA Resuscitation Characteristics According to Time Period for Age 18-64 Years **eTable 9.** OHCA Resuscitation Characteristics According to Time Period for Age ≥65 Years

Supplement 2.
Data Sharing Statement.
