# Supplementary material for: Temporal Patterns in Out-of-Hospital Cardiac Arrest Incidence and Outcome
Source: JAMA Cardiol. 2025 Jul 16;10(9):922–31. doi: 10.1001/jamacardio.2025.2247 (PMC12268528; doi:10.1001/jamacardio.2025.2247)
Supplement: Supplement 1. — eTable 1. OHCA Counts and Population Denominators According to Demographics and Initial Rhythm (King County, WA 2001-2020) eTable 2. Joinpoint Where Second Slope Term Improves Incidence vs Calendar Year Fit eTable 3. OHCA Resuscitation Characteristics According to Initial Rhythm and Time Period eTable 4. Relative Risk of Outcomes According to Time Period for Shockable Rhythm OHCA eTable 5. Relative Risk of Outcomes According to Time Period for Nonshockable Rhythms OHCA eTable 6. OHCA Resuscitation Characteristics According to Time Period for Home Location eTable 7. OHCA Resuscitation Characteristics According to Time Period for Public Location eTable 8. OHCA Resuscitation Characteristics According to Time Period for Age 18-64 Years eTable 9. OHCA Resuscitation Characteristics According to Time Period for Age ≥65 Years [file jamacardiol-e252247-s001.pdf]

Supplementary Online Content

McBride O, Poel A, Counts CR, et al. Temporal patterns in out-of-hospital cardiac arrest incidence and outcome. *JAMA Cardiol*. Published online July 16, 2025. doi:10.1001/jamacardio.2025.2247

eTable 1. OHCA Counts and Population Denominators According to Demographics and Initial Rhythm (King County, WA 2001-2020)

eTable 2. Joinpoint Where Second Slope Term Improves Incidence vs Calendar Year Fit

eTable 3. OHCA Resuscitation Characteristics According to Initial Rhythm and Time Period

eTable 4. Relative Risk of Outcomes According to Time Period for Shockable Rhythm OHCA

eTable 5. Relative Risk of Outcomes According to Time Period for Nonshockable Rhythms OHCA

eTable 6. OHCA Resuscitation Characteristics According to Time Period for Home Location

eTable 7. OHCA Resuscitation Characteristics According to Time Period for Public Location

eTable 8. OHCA Resuscitation Characteristics According to Time Period for Age 18-64 Years

eTable 9. OHCA Resuscitation Characteristics According to Time Period for Age ≥65 Years

This supplementary material has been provided by the authors to give readers additional information about their work.

© 2025 McBride O et al. *JAMA Cardiology*.

eTable 1. OHCA counts and population denominators according to demographics and initial rhythm (King County, WA 2001-2020)

	2001	2002	2003	2004	2005	2006	2007	2008	2009	2010	2011	2012	2013	2014	2015	2016	2017	2018	2019	2020
**Overall***	1209	1208	1135	1114	1181	1138	1170	1206	1192	1179	1198	1260	1264	1393	1261	1385	1310	1413	1425	1477
**Person-Years**	1362759	1381934	1393007	1405519	1418459	1443922	1465589	1483281	1499677	1517741	1527346	1539663	1561044	1590827	1620158	1663224	1703773	1737314	1769326	1799945
**Shockable**	390	361	328	304	306	326	323	342	299	296	299	321	283	347	300	331	327	331	306	322
**Person-Years**	1362759	1381934	1393007	1405519	1418459	1443922	1465589	1483281	1499677	1517741	1527346	1539663	1561044	1590827	1620158	1663224	1703773	1737314	1769326	1799945
**Non-Shockable**	815	845	797	807	871	800	840	857	879	873	885	925	968	1032	947	1044	971	1061	1100	1147
**Person-Years**	1362759	1381934	1393007	1405519	1418459	1443922	1465589	1483281	1499677	1517741	1527346	1539663	1561044	1590827	1620158	1663224	1703773	1737314	1769326	1799945
**Unknown Rhythm**	4	2	10	3	4	12	7	7	14	10	14	14	13	14	14	10	12	21	19	8
**Men***	772	769	713	718	727	736	728	768	739	750	745	784	807	854	809	894	864	933	927	957
**Person-Years**	672717	682739	688607	695118	701855	714817	725912	735000	743383	750215	755594	762413	773655	788943	804080	825843	846481	864205	880831	896549
**Women***	437	439	422	396	454	402	442	438	453	429	453	476	457	539	452	491	446	480	498	520
**Person-Years**	690042	699195	704400	710401	716604	729105	739677	748281	756294	767526	771752	777250	787389	801884	816078	837381	857292	873109	888495	903396
**Age <=64 years***	510	521	495	529	512	538	583	559	534	573	557	630	606	710	613	679	676	721	721	750
**Person-Years**	1180195	1198461	1208573	1220261	1232014	1254066	1271890	1284583	1295503	1307062	1310730	1311876	1323268	1344891	1366007	1403237	1437460	1471019	1495251	1517482
**Age >= 65 years***	699	687	640	585	669	600	587	647	658	606	641	630	658	683	648	706	634	692	704	727
**Person-Years**	182564	183473	184434	185258	186445	189856	193699	198698	204174	210679	216616	227787	237776	245936	254151	259987	266313	266295	274075	282463
**Shockable**																				
**18-64 years**																				
**Women**	50	38	32	35	35	38	42	37	31	23	28	42	39	45	37	41	39	35	38	44
**Person-Years**	583363	592260	597273	603143	608978	619933	628748	634985	640268	648038	649480	649459	654457	664561	674388	692137	708343	724047	735258	745697
**Men**	136	134	132	146	111	142	144	155	127	134	141	135	121	143	128	138	138	143	131	125
**Person-Years**	596832	606201	611300	617118	623036	634133	643142	649598	655235	659024	661250	662417	668811	680330	691619	711100	729117	746972	759993	771785
**>=65 years**																				
**Women**	60	59	53	31	49	41	33	34	38	39	33	44	27	41	28	34	36	34	28	27
**Person-Years**	106679	106935	107127	107258	107626	109172	110929	113296	116026	119488	122272	127791	132932	137323	141690	145244	148949	149062	153237	157699
**Men**	144	130	111	92	111	105	104	116	103	100	97	100	96	118	107	118	114	119	109	126
**Person-Years**	75885	76538	77307	78000	78819	80684	82770	85402	88148	91191	94344	99996	104844	108613	112461	114743	117364	117233	120838	124764
**Non-Shockable**																				
**18-64 years**																				
**Women**	110	133	115	132	120	122	149	143	156	143	150	178	155	205	164	164	181	194	195	202
**Person-Years**	583363	592260	597273	603143	608978	619933	628748	634985	640268	648038	649480	649459	654457	664561	674388	692137	708343	724047	735258	745697
**Men**	212	216	209	215	242	233	246	224	213	267	232	268	285	311	278	332	316	337	347	377
**Person-Years**	596832	606201	611300	617118	623036	634133	643142	649598	655235	659024	661250	662417	668811	680330	691619	711100	729117	746972	759993	771785
**>=65 years**																				
**Women**	215	209	220	198	250	195	215	221	223	220	235	204	230	240	218	246	187	208	230	245
**Person-Years**	106679	106935	107127	107258	107626	109172	110929	113296	116026	119488	122272	127791	132932	137323	141690	145244	148949	149062	153237	157699
**Men**	278	287	253	262	259	250	230	269	287	243	268	275	298	276	287	302	287	322	328	323
**Person-Years**	75885	76538	77307	78000	78819	80684	82770	85402	88148	91191	94344	99996	104844	108613	112461	114743	117364	117233	120838	124764

***includes unknown rhythms**

© 2025 McBride O et al. *JAMA Cardiology*.

eTable 2. Joinpoint where second slope term improves incidence versus calendar year fit

	Avg Annualized Change, %	95% CI
**Overall**		
2001-2020 **(**single linear term)	-0.5%	-0.9%,0.0%
Additional slope term improves fit		
2001-2004	*-3.6%*	*-8.1%, -0.3%*
2004-2020	0.1%	-0.3%, 1.7%
**Shockable**		
2001-2020 **(**single linear term)	-2.3%	-2.9%,-1.5%
Additional slope term improves fit		
2001-2004	*-7.70%*	*-14.2%,-2.0%*
2004-2020	-1.30%	-2.0%,1.8%
**Male Sex**		
2001-2020 **(**single linear term)	-0.5%	-0.8%, 0.1%
Additional slope term improves fit		
2001-2004	-4.4%	-8.5%, -0.4%
2004-2020	0.3%	-0.1,1.6

© 2025 McBride O et al. *JAMA Cardiology*.

eTable 3. OHCA resuscitation characteristics according to initial rhythm and time period

Characteristics	2001-2020		2001-2005		2006-2010		2011-2015		2016-2020	
	Shockable n = 6442	Nonshockable n = 18464	Shockable n = 1689	Nonshockable n = 4135	Shockable n = 1586	Nonshockable n = 4249	Shockable n = 1550	Nonshockable n = 4757	Shockable n = 1617	Nonshockable n = 5323
**Age, median (25th-75th %)**	63 (53-74)	66 (53-80)	64 (53-77)	70 (54-81)	62 (52-74)	68 (54-81)	62 (52-73)	66 (53-80)	63 (54-73)	65 (52-76)
**Female, n (%)**	1518 (23.6)	7520 (40.7)	442 (26.2)	1702 (41.2)	356 (22.4)	1787 (42.1)	364 (23.5)	1979 (41.6)	356 (22.0)	2052 (38.6)
**Cardiac Etiology, n (%)**	5943 (92.3)	10793 (58.5)	1579 (93.5)	2651 (64.1)	1471 (92.7)	2561 (60.3)	1186 (76.5)	2816 (59.2)	1453 (89.9)	2765 (51.9)
Location of Arrest										
**Home, n (%)**	3438 (53.4)	12278 (66.5)	928 (55.0)	2730 (66.0)	832 (52.5)	2767 (65.1)	793 (51.2)	3182 (66.9)	885 (54.7)	3599 (67.6)
**Public Indoor/Outdoor, n (%)**	2252 (34.9)	2209 (12.0)	521 (30.8)	415 (10.0)	566 (35.7)	450 (10.6)	594 (38.3)	606 (12.7)	571 (35.3)	738 (13.9)
**Healthcare Facility, n (%)**	181 (2.8)	245 (1.3)	42 (2.5)	45 (1.1)	40 (2.5)	54 (1.3)	40 (2.6)	47 (1.0)	59 (3.6)	99 (1.9)
**Other, n (%)**	571 (8.9)	3732 (20.2)	198 (11.7)	945 (22.9)	148 (9.3)	978 (23.0)	123 (7.9)	922 (19.4)	102 (6.3)	887 (16.7)
Witnessed										
**Bystander Witnessed, n (%)**	4291 (66.6)	5810 (31.5)	1096 (64.9)	1207 (29.1)	1029 (64.9)	1266 (29.8)	1054 (68.0)	1563 (32.9)	1112 (68.8)	1774 (33.3)
**EMS Witnessed, n (%)**	682 (10.6)	2182 (11.8)	196 (11.6)	432 (10.4)	179 (11.3)	501 (11.8)	140 (9.0)	559 (11.8)	167 (10.3)	690 (13.0)
**Bystander CPR, n (%)[a]**	4184 (72.6)	10197 (62.6)	971 (65.0)	1910 (51.2)	957 (68.0)	2198 (58.6)	1068 (75.7)	2770 (66.0)	1188 (81.9)	3319 (71.6)
Non-EMS AED Application[a]										
**AED Application, n (%)**	662 (11.5)	701 (4.3)	83 (5.6)	28 (0.8)	124 (7.8)	75 (1.8)	201 (14.3)	188 (4.5)	254 (17.5)	410 (8.8)
**AED by Law Enforcement, n (%)[b]**	165 (2.9)	399 (2.5)	-	-	-	1 (0.0)	57 (4.0)	103 (2.5)	108 (7.4)	295 (6.4)
**AED by Non-Law Enforcement, n (%)[b]**	274 (4.8)	189 (1.2)	-	-	23 (1.6)	19 (0.5)	106 (7.5)	57 (1.4)	145 (10.0)	113 (2.4)
**Arrest Occurred Prior to EMS Arrival, n (%)**	5758 (89.4)	16265 (88.1)	1493 (88.4)	3700 (89.5)	1406 (88.7)	3746 (88.2)	1410 (91.0)	4189 (88.1)	1449 (89.6)	4630 (87.0)
**BLS Response Time, median (25th-75th %)[c]**	5.0 (4.0-6.4)	5.1 (4.0-6.5)	5.0 (4.0-6.0)	5.0 (4.0-6.2)	5.0 (4.0-6.0)	5.0 (4.0-6.0)	5.0 (4.0-6.1)	5.0 (4.0-6.3)	5.5 (4.4-6.8)	5.6 (4.4-6.9)
**ALS Response Time, median (25th-75th %)[c]**	9.0 (6.9-12.0)	9.1 (7.0-12.4)	9.0 (6.9-12.0)	9.0 (7.0-12.0)	9.0 (6.9-12.0)	9.0 (6.9-12.0)	8.7 (6.8-11.6)	9.0 (6.7-12.0)	9.5 (7.1-12.3)	9.8 (7.4-13.1)
Prehospital Interventions										
**Advanced Airway, n (%)**	5892 (91.5)	15373 (83.3)	1577 (93.4)	3295 (79.7)	1479 (93.3)	3537 (83.2)	1409 (90.9)	3949 (83.0)	1427 (88.3)	4592 (86.3)
**Epinephrine Use, n (%)**	4535 (70.4)	14187 (76.8)	1096 (64.9)	2729 (66.0)	1226 (77.3)	3310 (77.9)	1097 (70.8)	3776 (79.4)	1116 (69.0)	4372 (82.1)
Prehospital Outcomes										
**Hospital Admission, n (%)**	4036 (62.7)	5987 (32.4)	976 (57.8)	1116 (27.0)	965 (60.8)	1383 (32.5)	1011 (65.2)	1634 (34.3)	1084 (67.0)	1854 (34.8)
Hospital Interventions[d]										
**Cooling, n (%)**	1923 (47.6)	2061 (34.4)	112 (11.5)	102 (9.1)	632 (65.5)	532 (38.5)	463 (45.8)	492 (30.1)	716 (66.1)	935 (50.4)
**Angiography, n (%)**	2387 (59.1)	415 (6.9)	403 (41.3)	55 (4.9)	623 (64.6)	109 (7.9)	497 (49.2)	88 (5.4)	864 (79.7)	163 (8.8)
**PCI, n (%)**	1251 (31.0)	144 (2.4)	248 (35.4)	15 (1.3)	297 (30.8)	44 (3.2)	248 (24.5)	32 (2.0)	458 (42.8)	53 (2.9)
Overall Outcomes										
**Survival to Hospital Discharge, n (%)**	2744 (42.6)	1625 (8.8)	591 (35.0)	265 (6.4)	652 (41.1)	356 (8.4)	733 (47.3)	468 (9.8)	768 (47.5)	536 (10.1)
**Survival w/ CPC ≤ 2, n (%)**	2498 (38.8)	1278 (6.9)	503 (29.8)	196 (4.7)	583 (36.8)	266 (6.3)	689 (44.5)	368 (7.7)	723 (44.7)	448 (8.4)
**Survival w/ CPC ≤ 2 among survivors, n (%)**	2498 (91.0)	1278 (78.6)	503 (85.1)	196 (74.8)	583 (89.6)	266 (74.9)	689 (94.0)	368 (79.5)	723 (94.3)	448 (84.2)
**Survivor Incidence (per 100,000 p-y)**	8.9	5.3	8.5	3.8	8.8	4.8	9.4	6.0	8.9	6.2
**Survivor Incidence CPC ≤ 2 (per100,000 p-y)**	8.1	4.1	7.2	2.8	7.9	3.6	8.8	4.7	8.3	5.2

a-Only patients who arrested before EMS arrival were eligible for bystander CPR and non-EMS AED application

b-AED application stratified by law enforcement versus public access information available as of 2007

c Response time is from 911 call to BLS/ALS arrival at scene

d- Denominator is cases with hospital admission

e- Person years is abbreviated p-y

© 2025 McBride O et al. *JAMA Cardiology*.

eTable 4. Relative risk of outcome according to time period for shockable rhythm OHCA

	Model 1 (Crude)			Model 2[a]			Model 3[b]		
Time Period	% (Crude)	RR (95% CI)	P value for trend	% (Adjusted)	RR (95% CI)	P value for trend	% (Adjusted)	RR (95% CI)	P value for trend
Overall **- Survival to hospital discharge**									
2001-2005	35.0	Ref.	<0.001	35.0	Ref.	<0.001	35.0	Ref.	<0.001
2006-2010	41.1	1.17 (1.08, 1.28)		40.3	1.15 (1.05, 1.25)		39.9	1.14 (1.05, 1.24)	
2011-2015	47.3	1.35 (1.24, 1.47)		45.9	1.31 (1.21, 1.42)		45.2	1.29 (1.19, 1.40)	
2016-2020	47.5	1.36 (1.25, 1.47)		46.2	1.32 (1.22, 1.44)		45.5	1.30 (1.20, 1.40)	
Overall **– Survival to hospital discharge with CPC 1-2**									
2001-2005	29.8	Ref.	<0.001	29.8	Ref.	<0.001	29.8	Ref.	<0.001
2006-2010	36.8	1.23 (1.12, 1.36)		35.8	1.20 (1.09, 1.33)		35.5	1.19 (1.09, 1.31)	
2011-2015	44.5	1.49 (1.36, 1.64)		42.9	1.44 (1.32, 1.58)		42.0	1.41 (1.29, 1.54)	
2016-2020	44.7	1.50 (1.37, 1.64)		43.5	1.46 (1.33, 1.60)		42.3	1.42 (1.30, 1.55)	
Prehospital Outcome **– Survival to hospital admission**									
2001-2005	57.8	Ref.	<0.001	57.8	Ref.	<0.001	57.8	Ref.	<0.001
2006-2010	60.8	1.05 (0.99, 1.11)		60.7	1.05 (0.99, 1.11)		60.7	1.05 (0.99, 1.11)	
2011-2015	65.2	1.13 (1.07, 1.19)		64.7	1.12 (1.06, 1.19)		64.2	1.11 (1.05, 1.17)	
2016-2020	67.0	1.16 (1.10, 1.22)		67.0	1.16 (1.10, 1.22)		65.9	1.14 (1.08, 1.20)	
In-Hospital Outcome **– Survival with CPC 1 or 2 among those admitted to hospital**									
2001-2005	51.5	Ref.	<0.001	51.5	Ref.	<0.001	51.5	Ref.	<0.001
2006-2010	60.4	1.13 (1.04, 1.22)		56.7	1.10 (1.01, 1.19)		56.7	1.10 (1.02, 1.19)	
2011-2015	68.2	1.30 (1.20, 1.40)		63.9	1.24 (1.15, 1.34)		64.4	1.25 (1.16, 1.34)	
2016-2020	66.7	1.26 (1.17, 1.36)		62.8	1.22 (1.13, 1.31)		62.3	1.21 (1.13, 1.30)	

a *– Model 2 adjusted for age and sex*

b *– Model 3 adjusted for age, sex, initial rhythm, witnessed status, and location of arrest*

© 2025 McBride O et al. *JAMA Cardiology*.

eTable 5. Relative risk of outcome according to time period for non-shockable rhythms OHCA

	Model 1 (Crude)			Model 2[a]			Model 3[b]		
**Time Period**	**% (Crude)**	**RR (95% CI)**	**P value for trend**	**% (Adjusted)**	**RR (95% CI)**	**P value for trend**	**% (Adjusted)**	**RR (95% CI)**	**P value for trend**
***Overall* - Survival to hospital discharge**									
2001-2005	6.4	Ref.	<0.001	6.4	Ref.	<0.001	6.4	Ref.	<0.001
2006-2010	8.4	1.31 (1.12, 1.52)		8.3	1.30 (1.11, 1.51)		8.1	1.26 (1.09, 1.47)	
2011-2015	9.8	1.54 (1.33, 1.77)		9.6	1.50 (1.29, 1.73)		8.8	1.38 (1.20, 1.60)	
2016-2020	10.1	1.57 (1.36, 1.81)		9.5	1.48 (1.29, 1.71)		8.3	1.30 (1.14, 1.50)	
***Overall* – Survival to hospital discharge with CPC 1-2**									
2001-2005	4.7	Ref.	<0.001	4.7	Ref.	<0.001	4.7	Ref.	<0.001
2006-2010	6.3	1.32 (1.10, 1.58)		6.2	1.31 (1.09, 1.57)		6.0	1.28 (1.07, 1.52)	
2011-2015	7.7	1.63 (1.38, 1.93)		7.4	1.58 (1.34, 1.87)		6.9	1.46 (1.23, 1.72)	
2016-2020	8.4	1.78 (1.51, 2.09)		7.8	1.66 (1.41, 1.95)		6.7	1.43 (1.22, 1.68)	
***Prehospital Outcome* – Survival to hospital admission**									
2001-2005	27.0	Ref.	<0.001	27.0	Ref.	<0.001	27.0	Ref.	<0.001
2006-2010	32.5	1.21 (1.13, 1.29)		32.4	1.20 (1.13, 1.29)		31.9	1.18 (1.11, 1.26)	
2011-2015	34.3	1.27 (1.19, 1.36)		34.0	1.26 (1.19, 1.35)		32.7	1.21 (1.14, 1.29)	
2016-2020	34.8	1.29 (1.21, 1.37)		34.6	1.28 (1.20, 1.36)		32.4	1.20 (1.13, 1.27)	
***In-Hospital Outcome* – Survival with CPC 1 or 2 among those admitted to hospital**									
2001-2005	17.6	Ref.	<0.001	Ref.	Ref.	<0.001	Ref.	Ref.	0.005
2006-2010	19.2	1.12 (0.94, 1.33)		19.5	1.11 (0.94, 1.32)		19.7	1.12 (0.95, 1.33)	
2011-2015	22.5	1.30 (1.10, 1.52)		22.2	1.26 (1.07, 1.47)		21.6	1.23 (1.05, 1.44)	
2016-2020	24.2	1.38 (1.18, 1.61)		22.7	1.29 (1.11, 1.51)		21.5	1.22 (1.04, 1.42)	

a *– Model 2 adjusted for age and sex*

b *– Model 3 adjusted for age, sex, initial rhythm, witnessed status, and location of arrest*

© 2025 McBride O et al. *JAMA Cardiology*.

eTable 6. OHCA resuscitation characteristics by time period: home location

Characteristics	Incident Years											
	2001-2005			2006-2010			2011-2015			2016-2020		
	Overall n = 3668	Shockable n = 928	Nonshockable n = 2730	Overall n = 3629	Shockable n = 832	Nonshockable n = 2767	Overall n = 4021	Shockable n = 793	Nonshockable n = 3182	Overall n = 4528	Shockable n = 885	Nonshockable n = 3599
**Age, median (25th-75th %)**	68 (54-79)	66 (55-78)	69 (53-80)	66 (53-79)	65 (54-75)	66 (53-80)	65 (53-78)	64 (54-75)	65 (53-79)	65 (53-76)	65 (55-74)	65 (53-76)
**Female, n (%)**	1425 (38.8)	272 (29.3)	1151 (42.2)	1402 (38.6)	213 (25.6)	1176 (42.5)	1611 (40.1)	218 (27.5)	1371 (43.1)	1656 (36.6)	208 (23.5)	1432 (39.8)
**Cardiac Etiology, n (%)**	2585 (70.5)	858 (92.5)	1718 (62.9)	2461 (67.8)	763 (91.7)	1681 (60.8)	2595 (64.5)	575 (72.5)	1839 (57.8)	2674 (59.1)	783 (88.5)	1866 (51.8)
Witnessed												
**Bystander Witnessed, n (%)**	1347 (36.7)	550 (59.3)	795 (29.1)	1304 (35.9)	489 (58.8)	805 (29.1)	1528 (38.0)	517 (65.2)	998 (31.4)	1768 (39.0)	579 (65.4)	1182 (32.8)
**EMS Witnessed, n (%)**	419 (11.4)	123 (13.3)	294 (10.8)	457 (12.6)	123 (14.8)	329 (11.9)	472 (11.7)	83 (10.5)	380 (11.9)	561 (12.4)	98 (11.1)	456 (12.7)
**Bystander CPR, n (%)**[a]	1559 (48.0)	476 (59.1)	1080 (44.3)	1768 (55.7)	452 (63.8)	1304 (53.5)	2278 (64.2)	520 (73.2)	1740 (62.1)	2797 (70.5)	628 (80.0)	2151 (68.4)
Non-EMS AED Application[a]												
**AED Application, n (%)**	17 (0.5)	9 (1.0)	8 (0.3)	19 (0.6)	6 (0.8)	13 (0.5)	122 (3.4)	45 (6.3)	77 (2.7)	260 (6.6)	55 (7.0)	200 (6.4)
**AED by Law Enforcement, n (%)**[b]	-	-	-		-	-	89 (2.5)	34 (4.8)	55 (2.0)	252 (6.4)	54 (6.9)	194 (6.2)
**AED by Non-Law Enforcement, n (%)**[b]	-	-	-	1 (0.0)	-	1 (0.0)	7 (0.3)	3 (0.4)	4 (0.1)	8 (0.2)	1 (0.1)	6 (0.2)
**Arrest Occurred Prior to EMS Arrival, n (%)**	3247 (88.5)	805 (86.7)	2434 (89.2)	3172 (87.4)	709 (85.2)	2438 (88.1)	3545 (88.2)	710 (89.5)	2798 (87.9)	3964 (87.5)	786 (88.8)	3141 (87.3)
**BLS Resp Time, median (25th-75th %)**[c]	5.3 (4.1-7.0)	5.3 (4.0-7.0)	5.3 (4.1-7.0)	5.0 (4.1-6.0)	5.0 (4.0-6.6)	5.0 (4.1-6.6)	5.2 (4.1-6.5)	5.2 (4.1-6.6)	5.2 (4.1-6.5)	5.7 (4.7-7.1)	5.8 (4.7-7.1)	5.7 (4.6-7.1)
**ALS Resp Time, median (25th-75th %)**[c]	9.7 (7.0-12.8)	9.0 (7.0-12.3)	10.0 (7.0-12.8)	9.2 (7.0-12.9)	9.0 (7.0-12.0)	9.3 (7.0-13.0)	9.1 (7.0-12.1)	9.0 (7.0-11.7)	9.1 (7.0-12.3)	10.1 (7.6-13.2)	9.8 (7.5-12.6)	10.1 (7.6-13.3)
Prehospital Interventions												
**Advanced Airway, n (%)**	3056 (83.3)	870 (93.8)	2179 (79.8)	3128 (86.2)	782 (94.0)	2332 (84.3)	3403 (84.6)	728 (91.8)	2651 (83.3)	3935 (86.9)	809 (91.4)	3111 (86.4)
**Epinephrine Use, n (%)**	2423 (66.1)	626 (67.5)	1793 (65.7)	2852 (78.6)	667 (80.2)	2177 (78.7)	3117 (77.5)	578 (72.9)	2525 (79.4)	3626 (80.1)	655 (74.0)	2959 (82.2)
Prehospital Outcomes												
**Hospital Admission, n (%)**	1233 (33.6)	511 (55.1)	721 (26.4)	1382 (38.1)	502 (60.3)	865 (31.3)	1601 (39.8)	504 (63.6)	1071 (33.7)	1782 (39.4)	563 (63.6)	1204 (33.5)
Hospital Interventions[d]												
**Cooling, n (%)**	89 (7.2)	37 (7.2)	52 (7.2)	660 (47.8)	335 (66.7)	320 (37.0)	807 (50.4)	349 (69.2)	454 (42.4)	1025 (57.5)	396 (70.3)	625 (51.9)
**Angiography, n (%)**	266 (21.6)	223 (43.6)	43 (6.0)	356 (25.8)	284 (56.6)	71 (8.2)	452 (28.2)	356 (70.6)	94 (8.8)	519 (29.1)	416 (73.9)	102 (8.5)
**PCI, n (%)**	155 (12.6)	140 (27.4)	15 (2.1)	164 (11.9)	139 (27.7)	25 (2.9)	214 (13.4)	178 (35.3)	36 (3.4)	259 (14.5)	225 (40.0)	34 (2.8)
Overall Outcomes												
**Survival to Hospital Discharge, n (%)**	448 (12.2)	277 (29.8)	169 (6.2)	533 (14.7)	315 (37.9)	210 (7.6)	660 (16.4)	342 (43.1)	295 (9.3)	691 (15.3)	362 (40.9)	315 (8.8)
**Survival with CPC ≤ 2, n (%)**	363 (9.9)	234 (25.2)	129 (4.7)	445 (12.3)	274 (32.9)	163 (5.9)	579 (14.4)	316 (39.8)	244 (7.7)	616 (13.6)	338 (38.2)	268 (7.4)
**Survival with CPC ≤ 2 among survivors, n (%)**	363 (81.0)	234 (84.5)	129 (76.3)	445 (83.5)	274 (87.0)	163 (77.6)	579 (87.7)	316 (92.4)	244 (82.7)	616 (89.1)	338 (93.4)	268 (85.1)
**Survivor Incidence (per 100,000 person-years)**	6.4	4.0	2.4	7.2	4.3	2.8	8.4	4.4	3.8	8.0	4.2	3.6
**Survivor Incidence with CPC ≤ 2 (per 100,000 person-years)**	5.2	3.4	1.9	6.0	3.7	2.2	7.4	4.0	3.1	7.1	3.9	3.1

a – *Only patients who arrested before EMS arrival were eligible for bystander CPR and non-EMS AED application*
b – *AED application stratified by law enforcement versus public access information available as of 2007*
c – *Response time is from 911 call to BLS/ALS arrival at scene*
d – *Denominator is cases with hospital admission*

© 2025 McBride O et al. *JAMA Cardiology*.

eTable 7. OHCA resuscitation characteristics by time period: public location

Characteristics	Incident Years											
	2001-2005			2006-2010			2011-2015			2016-2020		
	Overall n = 943	Shockable n = 521	Nonshockable n = 415	Overall n = 1025	Shockable n = 566	Nonshockable n = 450	Overall n = 1210	Shockable n = 594	Nonshockable n = 606	Overall n = 1317	Shockable n = 571	Nonshockable n = 738
**Age, median (25th-75th %)**	58 (48-70)	60 (51-70)	56 (45-70)	59 (49-69)	59 (51-68)	58 (46-72)	58 (47-68)	60 (50-68)	56 (45-68)	58 (47-67)	60 (52-69)	55 (41-66)
**Female, n (%)**	188 (19.9)	93 (17.9)	93 (22.4)	199 (19.4)	84 (14.8)	112 (24.9)	246 (20.3)	92 (15.5)	153 (25.2)	289 (21.9)	97 (17.0)	188 (25.5)
**Cardiac Etiology, n (%)**	790 (83.8)	509 (97.7)	277 (66.7)	813 (79.3)	546 (96.5)	262 (58.2)	929 (76.8)	502 (84.5)	353 (58.3)	891 (67.7)	533 (93.3)	358 (48.5)
Witnessed												
**Bystander Witnessed, n (%)**	555 (41.1)	395 (75.8)	159 (38.3)	588 (57.4)	427 (75.4)	157 (34.9)	701 (57.9)	445 (74.9)	248 (40.9)	735 (55.8)	443 (77.6)	288 (39.0)
**EMS Witnessed, n (%)**	95 (10.1)	47 (9.0)	46 (11.1)	107 (10.4)	37 (6.5)	70 (15.6)	110 (9.1)	38 (6.4)	71 (11.7)	128 (9.7)	33 (5.8)	93 (12.6)
**Bystander CPR, n (%)**[a]	486 (57.3)	329 (69.4)	155 (42.0)	569 (62.0)	370 (69.9)	193 (50.8)	745 (67.7)	424 (76.3)	315 (58.9)	892 (75.0)	446 (82.9)	442 (68.5)
Non-EMS AED Application[a]												
**AED Application, n (%)**	62 (7.3)	49 (10.3)	12 (3.3)	124 (13.5)	85 (16.1)	39 (10.3)	187 (17.0)	123 (22.1)	62 (11.6)	278 (23.4)	155 (28.8)	122 (18.9)
**AED by Law Enforcement, n (%)**[b]	-	-	-	1 (0.2)	-	1 (0.3)	49 (4.5)	19 (3.4)	30 (5.6)	121 (10.2)	47 (8.7)	73 (11.3)
**AED Non-Law Enforcement, n (%)**[b]	-	-	-	32 (3.5)	20 (3.8)	12 (3.2)	111 (10.1)	84 (15.1)	25 (4.7)	156 (13.1)	108 (20.1)	48 (7.4)
**Arrest Prior to EMS Arrival, n (%)**	848 (89.9)	474 (91.0)	369 (88.9)	915 (89.3)	528 (93.3)	378 (84)	1096 (90.6)	556 (93.6)	531 (87.6)	1188 (90.2)	538 (94.2)	644 (87.3)
**BLS Resp Time, median (25th-75th %)**[c]	4.9 (3.9-6.0)	4.8 (3.8-6.0)	5.0 (4.0-6.1)	4.4 (3.6-6.0)	4.5 (3.7-6.0)	4.3 (3.5-5.8)	4.6 (3.7-6.0)	4.6 (3.7-6.0)	4.6 (3.7-6.0)	5.1 (4.0-6.5)	5.1 (4.1-6.3)	5.1 (4.0-6.6)
**ALS Resp Time, median (25th-75th %)**[c]	9.0 (6.1-12.0)	8.0 (6.0-11.4)	9.1 (6.3-12.8)	9.0 (6.2-12.0)	9.0 (6.2-12.0)	9.0 (6.5-12.9)	8.6 (6.4-12.0)	8.3 (6.3-12.8)	9.0 (6.6-12.5)	9.7 (7.1-13.2)	9.1 (6.9-12.0)	10.2 (7.3-13.8)
Prehospital Interventions												
**Advanced Airway, n (%)**	871 (92.4)	493 (94.6)	374 (90.1)	941 (91.8)	530 (93.6)	403 (89.6)	1099 (90.8)	543 (91.4)	549 (90.6)	1148 (87.2)	489 (85.6)	653 (88.5)
**Epinephrine Use, n (%)**	639 (67.8)	319 (61.2)	317 (76.4)	785 (76.6)	420 (74.2)	361 (80.2)	924 (76.4)	406 (68.4)	516 (85.1)	956 (72.6)	357 (62.5)	596 (80.8)
Prehospital Outcomes												
**Hospital admission, n (%)**	495 (52.5)	343 (65.8)	149 (35.9)	550 (53.7)	363 (64.1)	180 (40.0)	684 (56.5)	416 (70.0)	258 (42.6)	733 (55.7)	420 (73.6)	308 (41.7)
Hospital Interventions[d]												
**Cooling, n (%)**	101 (20.4)	67 (19.5)	32 (21.5)	303 (55.1)	222 (61.2)	80 (44.4)	385 (56.3)	252 (60.6)	132 (51.2)	416 (56.8)	246 (58.6)	167 (54.2)
**Angiography, n (%)**	221 (44.6)	210 (61.2)	11 (7.4)	279 (50.7)	259 (71.3)	20 (11.1)	359 (52.5)	332 (79.8)	27 (10.5)	387 (52.8)	356 (84.8)	31 (10.1)
**PCI, n (%)**	108 (21.8)	108 (31.5)	-	133 (24.2)	122 (33.6)	11 (6.1)	167 (24.4)	163 (39.2)	4 (1.6)	184 (25.1)	175 (41.7)	9 (2.9)
Overall Outcomes												
**Survival to Hospital Discharge, n (%)**	300 (31.8)	249 (47.8)	49 (11.8)	358 (34.9)	278 (49.1)	76 (16.9)	435 (36.0)	335 (56.4)	93 (15.3)	471 (35.8)	343 (60.1)	124 (16.8)
**Survival with CPC ≤ 2, n (%)**	253 (26.8)	220 (42.1)	32 (7.7)	321 (31.3)	258 (45.6)	59 (13.1)	410 (33.9)	324 (54.5)	80 (13.1)	439 (33.3)	326 (57.0)	109 (14.8)
**Survival with CPC ≤ 2 among survivors, n (%)**	253 (84.3)	220 (88.3)	32 (65.3)	321 (89.7)	258 (92.8)	59 (77.6)	410 (94.3)	324 (96.7)	80 (86.0)	439 (93.2)	326 (95.0)	109 (87.9)
**Survivor Incidence (per 100,000 person-years)**	4.3	3.6	0.7	4.8	3.8	1.0	5.5	4.3	1.2	5.4	4.0	1.4
**Survivor Incidence with CPC ≤ 2 (per 100,000 person-years)**	3.6	3.2	0.5	4.3	3.5	0.8	5.2	4.1	1.0	5.1	3.8	1.3

a – *Only patients who arrested before EMS arrival were eligible for bystander CPR and non-EMS AED application*
b – *AED application stratified by law enforcement versus public access information available as of 2007*
c – *Response time is from 911 call to BLS/ALS arrival at scene*
d – *Denominator is cases with hospital admission*

© 2025 McBride O et al. *JAMA Cardiology*.

eTable 8. OHCA resuscitation characteristics by time period: age 18-64 years

Characteristics	Incident Years											
	2001-2005			2006-2010			2011-2015			2016-2020		
	Overall n = 2567	Shockable n = 849	Nonshockable n = 1704	Overall n = 2787	Shockable n = 873	Nonshockable n = 1896	Overall n = 3116	Shockable n = 859	Nonshockable n = 2226	Overall n = 3547	Shockable n = 872	Nonshockable n = 2645
**Age, median (25th-75th %)**	52 (43-58)	53 (46-59)	51 (42-57)	52 (44-59)	53 (47-59)	52 (43-58)	53 (44-59)	54 (45-59)	52 (43-59)	53 (41-59)	54 (47-60)	52 (39-59)
**Female, n (%)**	803 (31.3)	190 (22.4)	610 (35.8)	890 (31.9)	171 (19.6)	713 (37.6)	1053 (33.8)	191 (22.2)	852 (38.3)	1144 (32.3)	197 (22.6)	936 (35.4)
**Cardiac Etiology, n (%)**	1661 (64.7)	791 (93.2)	860 (50.5)	1689 (60.6)	806 (92.3)	878 (46.3)	1804 (57.9)	785 (91.4)	1008 (45.3)	1787 (50.4)	754 (86.5)	1023 (38.7)
Location of Arrest												
**Home, n (%)**	1587 (61.8)	422 (49.7)	1161 (68.1)	1706 (61.2)	411 (47.1)	1284 (67.7)	1962 (63.0)	407 (47.4)	1532 (68.8)	2197 (61.9)	432 (49.5)	1748 (66.1)
**Public Indoor/Outdoor, n (%)**	619 (24.1)	330 (38.9)	282 (16.5)	673 (24.1)	376 (43.1)	293 (15.5)	804 (25.8)	381 (44.4)	415 (18.6)	902 (25.5)	362 (41.5)	535 (20.2)
**Healthcare Facility, n (%)**	44 (1.7)	19 (2.2)	24 (1.4)	39 (1.4)	19 (2.2)	20 (1.1)	36 (1.2)	18 (2.1)	18 (0.8)	80 (2.3)	34 (3.9)	44 (1.7)
**Other, n (%)**	316 (12.3)	78 (9.2)	236 (13.8)	369 (13.2)	67 (7.7)	299 (15.8)	313 (10.0)	53 (6.2)	260 (11.7)	367 (10.3)	43 (4.9)	318 (12.0)
Witnessed												
**Bystander Witnessed, n (%)**	959 (37.4)	561 (66.1)	395 (23.2)	1086 (39.0)	603 (69.1)	478 (25.2)	1225 (39.3)	592 (68.9)	621 (27.9)	1395 (39.3)	601 (68.9)	789 (29.8)
**EMS Witnessed, n (%)**	285 (11.1)	104 (12.2)	177 (10.4)	306 (11.0)	87 (10.0)	216 (11.4)	341 (10.9)	78 (9.1)	257 (11.5)	422 (11.9)	95 (10.9)	320 (12.1)
**Bystander CPR, n (%)[a]**	1318 (57.8)	502 (67.4)	809 (53.0)	1588 (64.0)	554 (70.5)	1023 (60.9)	1947 (70.2)	603 (77.2)	1330 (67.5)	2378 (76.1)	656 (84.4)	1708 (73.5)
***Non-EMS AED Application*[a]**												
**AED Application, n (%)**	63 (2.8)	49 (6.6)	12 (0.8)	123 (5.0)	80 (10.2)	43 (2.6)	218 (7.9)	113 (14.5)	104 (5.3)	413 (13.2)	144 (18.5)	263 (11.3)
**AED by Law Enforcement, n (%)[b]**	-	-	-	1 (0.0)	-	1 (0.1)	96 (3.5)	30 (3.8)	66 (3.4)	256 (8.2)	57 (7.3)	195 (8.4)
**AED Non-Law Enforcement, n (%)[b]**	-	-	-	32 (1.3)	19 (2.4)	13 (0.8)	91 (3.3)	64 (8.2)	26 (1.3)	154 (4.9)	86 (11.1)	66 (2.8)
**Arrest Prior to EMS Arrival, n (%)**	2280 (88.8)	745 (87.8)	1525 (89.5)	2479 (88.9)	786 (90.0)	1678 (88.5)	2771 (88.9)	781 (90.9)	1965 (88.3)	3125 (88.1)	777 (89.1)	2325 (87.9)
**BLS Resp Time, median (25th-75th %)[c]**	5.0 (4.0-6.4)	5.0 (4.0-6.0)	5.0 (4.0-6.8)	5.0 (4.0-6.0)	5.0 (4.0-6.0)	5.0 (4.0-6.0)	5.0 (4.0-6.3)	5.0 (3.9-6.1)	5.0 (4.0-6.3)	5.5 (4.4-6.9)	5.4 (4.4-6.9)	5.5 (4.4-7.0)
**ALS Resp Time, median (25th-75th %)[c]**	9.0 (7.0-12.0)	9.0 (7.0-12.0)	9.0 (7.0-12.0)	9.0 (6.9-12.0)	9.0 (7.0-12.0)	9.0 (6.9-12.2)	8.8 (6.7-11.8)	8.7 (6.8-11.5)	9.0 (6.7-12.0)	9.7 (7.3-13.0)	9.5 (7.1-12.7)	9.8 (7.4-13.1)
Prehospital Interventions												
**Advanced Airway, n (%)**	2198 (85.6)	796 (93.8)	1395 (81.9)	2426 (87.0)	818 (93.7)	1597 (84.2)	2681 (86.0)	785 (91.4)	1876 (84.3)	3049 (86.0)	768 (88.1)	2268 (85.7)
**Epinephrine Use, n (%)**	1725 (67.2)	548 (64.5)	1172 (68.8)	2184 (78.4)	671 (76.9)	1508 (79.5)	2405 (77.2)	599 (69.7)	1797 (80.7)	2751 (77.6)	584 (67.0)	2158 (81.6)
Prehospital Outcomes												
**Hospital Admission, n (%)**	978 (38.1)	508 (59.8)	466 (27.3)	1182 (42.4)	541 (62.0)	628 (33.1)	1418 (45.5)	587 (68.3)	806 (36.2)	1592 (44.9)	611 (70.1)	969 (36.6)
***Hospital Interventions*[d]**												
**Cooling, n (%)**	142 (14.5)	71 (14.0)	69 (14.8)	675 (57.1)	382 (70.6)	291 (46.3)	789 (55.6)	400 (68.1)	387 (48.0)	994 (62.4)	418 (68.4)	569 (58.7)
**Angiography, n (%)**	325 (33.2)	304 (59.8)	21 (4.5)	447 (37.8)	396 (73.2)	51 (8.1)	557 (39.3)	489 (83.3)	67 (8.3)	608 (38.2)	524 (85.8)	82 (8.5)
**PCI, n (%)**	177 (18.1)	173 (34.1)	4 (0.9)	216 (18.3)	195 (36.0)	21 (3.3)	262 (18.5)	246 (41.9)	16 (2.0)	295 (18.5)	269 (44.0)	25 (2.6)
Overall Outcomes												
**Survival to Hospital Discharge, n (%)**	482 (18.8)	355 (41.8)	125 (7.3)	603 (21.6)	410 (47.0)	183 (9.7)	783 (25.1)	479 (55.8)	285 (12.8)	857 (24.2)	497 (57.0)	351 (13.3)
**Survival with CPC ≤ 2, n (%)**	402 (15.7)	310 (36.5)	91 (5.3)	534 (19.2)	374 (42.8)	151 (8.0)	704 (22.6)	450 (52.4)	236 (10.6)	784 (22.1)	475 (54.5)	301 (11.4)
**Survival with CPC ≤ 2 among survivors, n (%)**	402 (83.4)	310 (87.3)	91 (72.8)	534 (88.6)	374 (91.2)	151 (82.5)	704 (90.0)	450 (93.9)	236 (82.8)	784 (91.5)	475 (95.6)	301 (85.8)
**Survivor Incidence (per 100,000 person-years)**	8.0	5.9	2.1	9.4	6.4	2.9	11.8	7.2	4.3	11.7	6.8	4.8
**Survivor Incidence with CPC ≤ 2 (per 100,000 person-years)**	6.7	5.1	1.5	8.3	5.8	2.4	10.6	6.8	3.5	10.7	6.5	4.1

a – *Only patients who arrested before EMS arrival were eligible for bystander CPR and non-EMS AED application*
b – *AED application stratified by law enforcement versus public access information available as of 2007*
c – *Response time is from 911 call to BLS/ALS arrival at scene*
d – *Denominator is cases with hospital admission*

© 2025 McBride O et al. *JAMA Cardiology*.

eTable 9. OHCA resuscitation characteristics by time period: age ≥65 years

Characteristics	Incident Years											
	2001-2005			2006-2010			2011-2015			2016-2020		
	Overall n = 3280	Shockable n = 840	Nonshockable n = 2431	Overall n = 3098	Shockable n = 713	Nonshockable n = 2353	Overall n = 3260	Shockable n = 691	Nonshockable n = 2531	Overall n = 3463	Shockable n = 745	Nonshockable n = 2678
**Age, median (IQR)**	79 (73-84)	77 (71-82)	79 (73-85)	79 (72-86)	75 (70-83)	80 (73-86)	78 (71-85)	74 (69-81)	79 (71-86)	76 (70-83)	74 (69-80)	76 (70-84)
**Female, n (%)**	1345 (41.0)	252 (30.0)	1092 (44.9)	1274 (41.1)	185 (25.9)	1074 (45.6)	1324 (40.6)	173 (25.0)	1127 (44.5)	1291 (37.3)	159 (21.3)	1116 (41.7)
**Cardiac Etiology, n (%)**	2587 (78.9)	788 (93.8)	1791 (73.7)	2372 (76.6)	665 (93.3)	1683 (71.5)	2493 (76.5)	655 (94.8)	1808 (71.4)	2467 (71.2)	699 (93.8)	1742 (65.0)
Location of Arrest												
**Home, n (%)**	2081 (63.4)	506 (60.2)	1569 (64.5)	1923 (62.1)	421 (59.0)	1483 (63.0)	2059 (63.2)	386 (55.9)	1650 (65.2)	2331 (67.3)	453 (60.8)	1851 (69.1)
**Public Indoor/Outdoor, n (%)**	324 (9.9)	191 (22.7)	133 (5.5)	352 (11.4)	190 (26.6)	157 (6.7)	406 (12.5)	213 (30.8)	191 (7.5)	415 (12.0)	209 (28.1)	203 (7.6)
**Healthcare Facility, n (%)**	44 (1.3)	23 (2.7)	21 (0.9)	55 (1.8)	21 (2.9)	34 (1.4)	51 (1.6)	22 (3.2)	29 (1.1)	80 (2.3)	25 (3.4)	55 (2.1)
**Other, n (%)**	831 (25.3)	120 (14.3)	708 (29.1)	768 (24.8)	81 (11.4)	679 (28.9)	744 (22.8)	70 (10.1)	661 (26.1)	636 (18.4)	58 (7.8)	569 (21.2)
Witnessed												
**Bystander Witnessed, n (%)[a]**	1348 (41.1)	535 (63.7)	812 (33.4)	1225 (39.5)	426 (59.7)	788 (33.5)	1418 (43.5)	462 (66.9)	942 (37.2)	1511 (43.6)	511 (68.6)	985 (36.8)
**EMS Witnessed, n (%)**	349 (10.6)	92 (11.0)	255 (10.5)	381 (12.3)	92 (12.9)	285 (12.1)	370 (11.3)	62 (9.0)	302 (11.9)	447 (12.9)	72 (9.7)	370 (13.8)
**Bystander CPR, n (%)**	1573 (53.7)	469 (62.7)	1101 (50.6)	1590 (58.5)	403 (64.9)	1175 (56.8)	1923 (66.5)	465 (73.9)	1440 (64.6)	2162 (71.7)	532 (79.0)	1611 (69.8)
Non-EMS AED Application[a]												
**AED Application, n (%)**	50 (1.7)	34 (4.5)	16 (0.7)	76 (2.8)	44 (7.1)	32 (1.5)	173 (6.0)	88 (14.0)	84 (3.8)	259 (8.6)	110 (16.3)	147 (6.4)
**AED by Law Enforcement, n (%)[b]**	-	-	-	-	-	-	64 (2.2)	27 (4.3)	37 (1.7)	153 (5.1)	51 (7.6)	100 (4.3)
**AED by Non-Law Enforcement, n (%)[b]**	-	-	-	10 (0.4)	4 (0.6)	6 (0.3)	74 (2.6)	42 (6.7)	31 (1.4)	106 (3.5)	59 (8.8)	47 (2.0)
**Arrest Prior to EMS Arrival, n (%)**	2930 (89.3)	748 (89.0)	2175 (89.5)	2716 (87.7)	620 (87.0)	2068 (87.9)	2885 (88.5)	629 (91.0)	2224 (87.9)	3012 (87.0)	672 (90.2)	2305 (86.1)
**BLS Response Time, median (IQR)[c]**	5.0 (4.0-6.0)	5.0 (4.0-6.0)	5.0 (4.0-6.0)	5.0 (4.0-6.0)	5.0 (4.0-6.0)	5.0 (4.0-6.0)	5.0 (4.0-6.2)	5.0 (4.0-6.3)	5.0 (4.0-6.2)	5.6 (4.4-6.9)	5.4 (4.4-6.8)	5.6 (4.4-6.9)
**ALS Response Time, median (IQR)[c]**	9.0 (6.7-12.0)	9.0 (6.0-11.1)	9.0 (6.9-12.0)	9.0 (6.7-12.0)	9.0 (6.8-12.0)	9.0 (6.8-12.0)	9.0 (6.8-11.9)	8.7 (6.8-11.8)	9.0 (6.9-12.0)	9.7 (7.4-12.9)	9.5 (7.0-12.0)	9.9 (7.4-13.1)
Prehospital Interventions												
**Advanced Airway, n (%)**	2690 (82.0)	781 (93.0)	1900 (78.2)	2619 (84.5)	661 (92.7)	1940 (82.4)	2714 (83.3)	624 (90.3)	2073 (81.9)	2998 (86.6)	659 (88.5)	2324 (86.8)
**Epinephrine Use, n (%)**	2109 (64.3)	548 (65.2)	1557 (64.0)	2368 (76.4)	555 (77.8)	1802 (76.6)	2488 (76.3)	498 (72.1)	1979 (78.2)	2758 (79.6)	532 (71.4)	2214 (82.7)
Prehospital Outcomes												
**Hospital Admission, n (%)**	1122 (34.2)	468 (55.7)	650 (26.7)	1193 (38.5)	424 (59.5)	755 (32.1)	1268 (38.9)	424 (61.4)	828 (32.7)	1371 (39.6)	473 (63.5)	885 (33.0)
Hospital Interventions[d]												
**Cooling, n (%)**	84 (7.5)	46 (9.8)	38 (5.8)	512 (42.9)	255 (60.1)	252 (33.4)	617 (48.6)	283 (66.7)	331 (40.0)	699 (51.0)	302 (63.8)	393 (44.4)
**Angiography, n (%)**	213 (19.0)	174 (37.2)	39 (6.0)	287 (24.1)	227 (53.5)	58 (7.7)	353 (27.8)	273 (64.4)	78 (9.4)	423 (30.9)	342 (72.3)	81 (9.2)
**PCI, n (%)**	116 (10.3)	104 (22.2)	12 (1.8)	126 (10.6)	102 (24.1)	23 (3.0)	164 (12.9)	135 (31.8)	29 (3.5)	217 (15.9)	189 (40.0)	28 (3.2)
Overall Outcomes												
**Survival to Hospital Discharge, n (%)**	377 (11.5)	236 (28.1)	140 (5.8)	421 (13.6)	242 (33.9)	173 (7.4)	449 (13.8)	254 (36.8)	183 (7.2)	465 (13.4)	271 (36.4)	185 (6.9)
**Survival with CPC ≤ 2, n (%)**	298 (9.1)	193 (23.0)	105 (4.3)	329 (10.6)	209 (29.3)	115 (4.9)	382 (11.7)	239 (34.6)	132 (5.2)	403 (11.6)	248 (33.3)	147 (5.5)
**Survival with CPC ≤ 2 among survivors, n (%)**	298 (79.0)	193 (81.8)	105 (75.0)	329 (78.1)	209 (86.4)	115 (66.5)	382 (85.1)	239 (94.1)	132 (72.1)	403 (86.7)	248 (91.5)	147 (79.5)
**Survivor Incidence (per 100,000 person-years)**	40.9	25.6	15.2	42.2	24.3	17.4	38.0	21.5	15.5	34.5	20.1	13.7
**Survivor Incidence with CPC ≤ 2 (per 100,000 person-years)**	32.3	20.9	11.4	33.0	21.0	11.5	32.3	20.2	11.2	29.9	18.4	10.9

a – *Only patients who arrested before EMS arrival were eligible for bystander CPR and non-EMS AED application*
b – *AED application stratified by law enforcement versus public access information available as of 2007*
c – *Response time is from 911 call to BLS/ALS arrival at scene*
d – *Denominator is cases with hospital admission*

© 2025 McBride O et al. *JAMA Cardiology*.
