# Supplementary material for: Temporal Patterns in Out-of-Hospital Cardiac Arrest Incidence and Outcome
Source: JAMA Cardiol. 2025 Jul 16;10(9):922–31. doi: 10.1001/jamacardio.2025.2247 (PMC12268528; doi:10.1001/jamacardio.2025.2247)
Supplement: Supplement 2. — Data Sharing Statement. [file jamacardiol-e252247-s002.pdf]

Data Sharing Statement

McBride. Temporal Patterns in Out-of-Hospital Cardiac Arrest Incidence and Outcome. *JAMA Cardiol*. Published July 16, 2025. doi:10.1001/jamacardio.2025.2247

Data
Data available: No

Additional Information
Explanation for why data not available: We have provided the data to generate incidence in the supplemental appendix. The detailed characteristic and outcome data about the cardiac arrest cohort is part of a protected quality improvement resource. The authors would be willing to consider reasonable requests to partner in research that would use this data resource.
